# Direct input of monitoring data into a mechanistic ecological model as a way to identify the phytoplankton growth-rate response to temperature variations

**DOI:** 10.1038/s41598-023-36950-3

**Published:** 2023-06-22

**Authors:** Alexander B. Medvinsky, Nailya I. Nurieva, Boris V. Adamovich, Nataly P. Radchikova, Alexey V. Rusakov

**Affiliations:** 1 Institute of Theoretical and Experimental Biophysics, Russian Academy of Sciences, Pushchino, Russia 142290; 2 Belarusian State University, 220010 Minsk, Belarus; 3 Moscow State University of Psychology and Education, Moscow, Russia 127051

**Keywords:** Ecology, Ecological modelling, Ecology, Ecological modelling

## Abstract

We present an approach (knowledge-and-data-driven, KDD, modeling) that allows us to get closer to understanding the processes that affect the dynamics of plankton communities. This approach, based on the use of time series obtained as a result of ecosystem monitoring, combines the key features of both the knowledge-driven modeling (mechanistic models) and data-driven (DD) modeling. Using a KDD model, we reveal the phytoplankton growth-rate fluctuations in the ecosystem of the Naroch Lakes and determine the degree of phase synchronization between fluctuations in the phytoplankton growth rate and temperature variations. More specifically, we estimate a numerical value of the phase locking index (*PLI*), which allows us to assess how temperature fluctuations affect the dynamics of phytoplankton growth rates. Since, within the framework of KDD modeling, we directly include the time series obtained as a result of field measurements in the model equations, the dynamics of the phytoplankton growth rate obtained from the KDD model reflect the behavior of the lake ecosystem as a whole, and *PLI* can be considered as a holistic parameter.

## Introduction

Phytoplankton as a primary producer plays an important role in the functioning of aquatic ecosystems^1,2^. The functioning of plankton communities depends on a plethora of biotic and abiotic factors. Among these factors, temperature plays a vital role. Since temperature affects the rates of many reactions including photosynthesis and respiration^3,4^, the phytoplankton biomass and growth can immediately respond to changes in temperature conditions^5^. Along with the direct influence of temperature on the growth rate of phytoplankton, temperature variations can also have an indirect effect on the dynamics of phytoplankton: for example, through the activity of zooplankton as the consumer of phytoplankton^6,7^ and through phase synchronization of oscillations of phytoplankton and bacterioplankton^8^.

In order to take into account the influence of various factors on plankton dynamics, mechanistic models describing the interaction of aquatic populations and the influence of abiotic processes (including temperature variations) on fluctuations in plankton abundance are often used^1,9^. Within the framework of this approach (the knowledge-driven, KD, modeling), the functions describing trophic interactions between populations, the mobility of organisms and, if necessary, the influence of abiotic factors on populations are assumed to be known^10^. The complex structure of inter-population interactions^11,12^ and the nonlinear nature of population and abiotic processes^1,13^ force researchers to reduce the mathematical description of ecological systems in order to make this description more readily understandable^14^.

In order to overcome the “curse of reductionism”^15^ and to be able to predict population dynamics, an attractor reconstruction method (empirical dynamic modeling) using time series obtained from field observations was proposed^16,17^. Another approach, virtual population analysis (VPA, including multi-species), aimed at overcoming reductionism, is widely used in fisheries science and is the most common method of determining the size of stocks in the past using mortality indicators^18^. In addition, an approach to mathematical modeling of natural systems has been developed (the data-driven, DD, modeling), in which a statistical inference (from e.g. artificial neural networks^19^ or genetic programming^20^) has been used to obtain previously unknown dependencies between system’s inputs and outputs from the available data (time series) to forecast future system’s outputs. At the same time, the real processes that determine the observed dynamics of natural systems in this case are usually not taken into account, and a formal mathematical description of the behavior of natural systems in the framework of the DD approach often does not make physical sense. The DD modeling is used only for the purpose of capturing the relationships between the pertinent input and output variables^21^.

Here, in order to assess to what extent temperature fluctuations are related to the growth rate of phytoplankton, we propose to expand the concept of DD modeling. Namely, we propose, as with DD modeling, to use the data obtained during the research of real systems. However, in order to study ecosystem processes, we intend, unlike what is proposed in the framework of DD modeling, to use the time series obtained during ecosystem monitoring in combination with mechanistic KD models. We have designated this approach to modeling ecosystem processes as the knowledge-and-data-driven, KDD, modeling. With the help of the KDD modeling, it becomes possible to study those dynamic regimes that directly reflect the features of a particular ecosystem, but which were not identified during monitoring of this ecosystem. In this article, we focus on the analysis of fluctuations in the growth rate of phytoplankton and the conjugacy of these fluctuations with temperature fluctuations using the example of the ecosystem of the Naroch Lakes (Belarus).

## Knowledge-and-data-driven (KDD) modeling as a method for revealing the dynamics of phytoplankton growth rate in a lake ecosystem

As is often assumed within the KD paradigm, population size changes occur, firstly, due to intra-population processes that depend on the population size and are also influenced by external factors (for example, temperature) and, secondly, due to inter-population interactions. Then a prey-predator model system can be given as follows:

1 $$\frac{1}{P\left( t \right)}\frac{\Delta P\left( t \right)}{{\Delta t}} = G\left( t \right) - f\left( t \right)Z\left( t \right)$$

2 $$\frac{1}{Z\left( t \right)}\frac{\Delta Z\left( t \right)}{{\Delta t}} = \beta f\left( t \right)P\left( t \right) - \alpha$$

Here* P*(*t*) and *Z*(*t*) are the abundance of prey and the abundance of predator, respectively; *t* is time. *G*(*t*) is the intrinsic prey growth rate, which can be influenced by external factors and can take positive, negative, or zero values; *f*(*t*) is the function that describes the intensity of predation and, in general, it may also depend on environmental factors; *α* and *β* are constants, *α* is the intensity of the decline in the predator abundance unrelated to trophic interactions between prey and predator, and *β* is the efficiency of turning prey into the predator abundance. Within the framework of the KD approach, the functions *G*(*t*) and *f*(*t*) are set analytically, i.e., in the form of mathematical formulas, and the solution of Eqs. (1) and (2), i.e., the functions $\widetilde{P}(t)$ and $\widetilde{Z}(t)$, turn out to be significantly dependent on the choice of the functions *G*(*t*) and *f*(*t*)^22,23^, as well as on the parameters of the model under study^14^.

**Figure 1:**
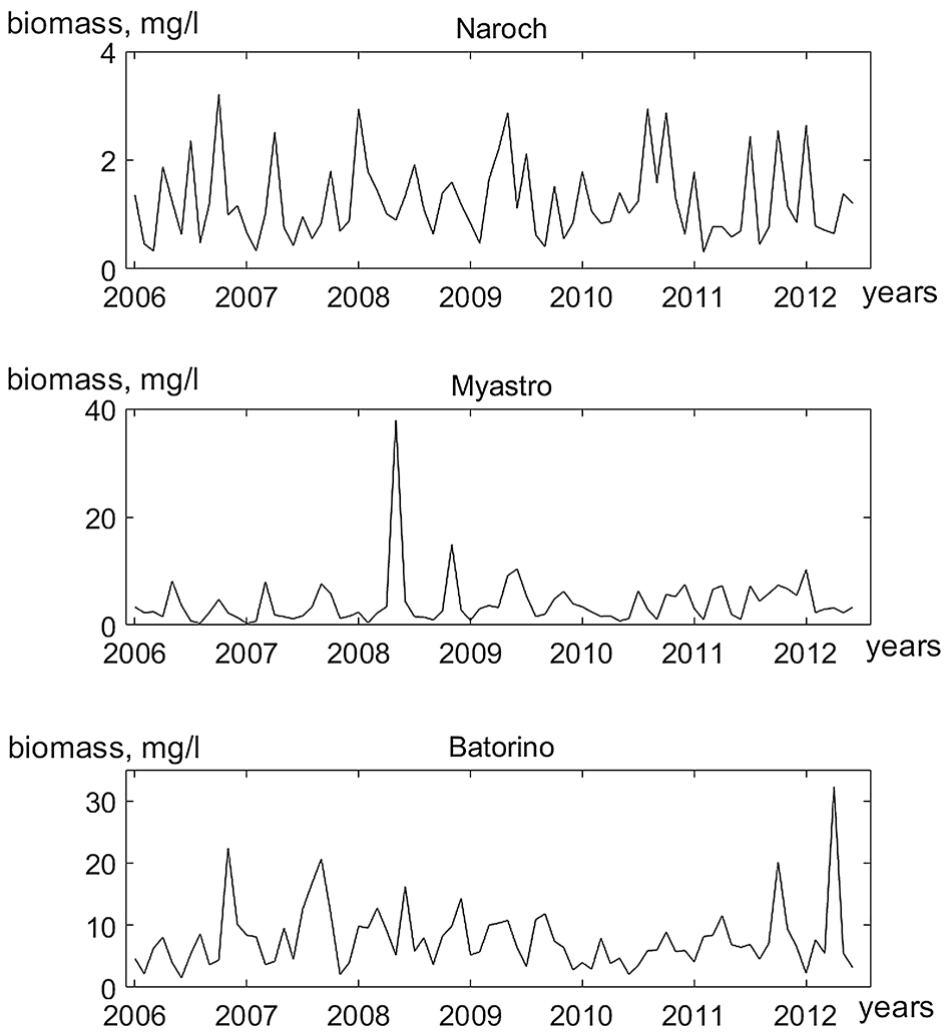
Time series plot of phytoplankton abundances in the Naroch Lakes (the time step corresponds to 1 month).

**Figure 2:**
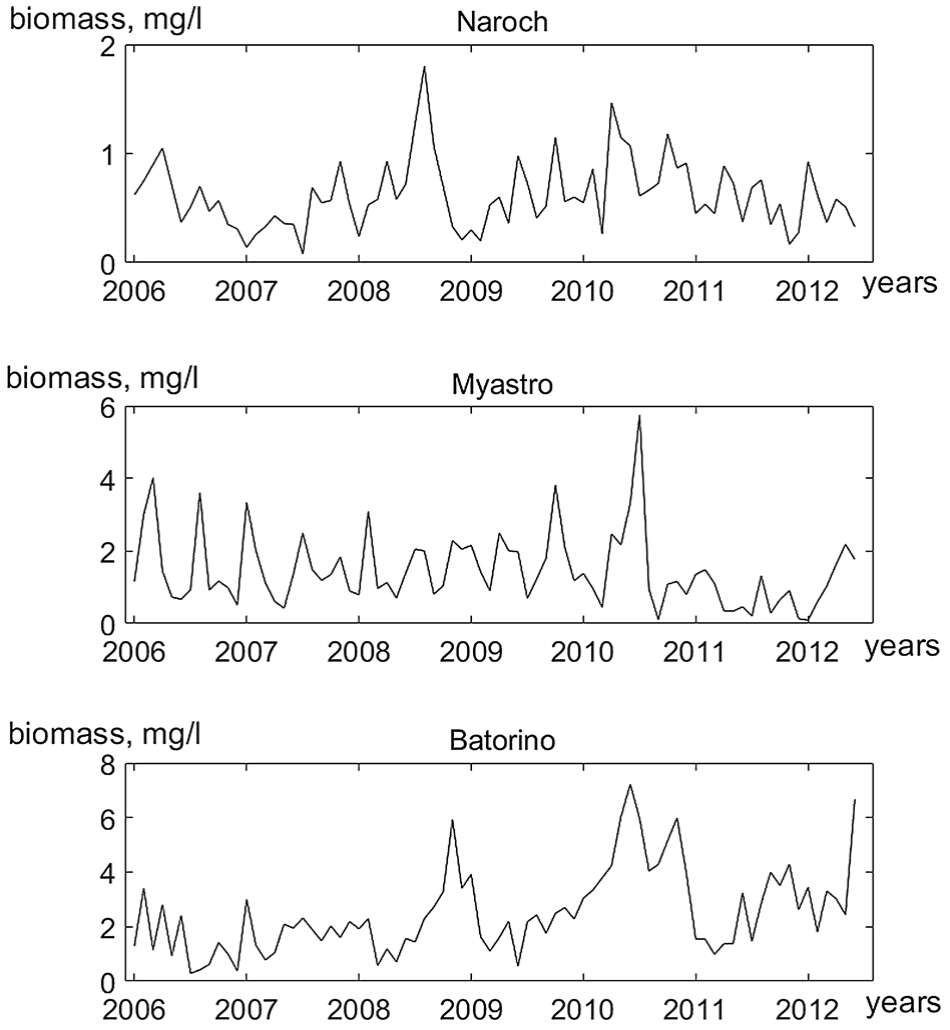
Time series plot of zooplankton abundances in the Naroch Lakes (the time step corresponds to 1 month).

In the context of the KDD modeling, it is the functions *P*(*t*) and *Z*(*t*) that are set initially. In our case, the functions *P*(*t*) (Fig. 1) and *Z*(*t*) (Fig. 2) are time series. These time series are the result of analyzing data from long-term monitoring of the Naroch Lakes ecosystem, which is situated in the Northwestern Belarus. It includes three lakes, Lake Batorino, Lake Myastro, and Lake Naroch, that are interconnected by channels. The main characteristics of the Naroch Lakes are given in^24^ (see also Supplementary Information 1). The plankton sampling, sample analysis, and the results of field measurements on the basis of which these time series are constructed are described in Supplementary Information. Now that the functions *P*(*t*) and *Z*(*t*) are given, it becomes possible to obtain fluctuations in the growth rate *G*(*t*) and in the trophic function *f*(*t*) (in the form of time series) from (1) and (2). Note that these fluctuations were not measured in the course of monitoring. In this paper, we focus on analyzing the dynamics of the growth rate of phytoplankton *G*(*t*) in each of the reservoirs of the the Naroch Lakes ecosystem.

## Relationship between phytoplankton growth rate and temperature

Taking into account Eqs. (1) and (2), and due to the discreteness of measurements, the result of which is the time series shown in Figs. 1 and 2, the growth rate of phytoplankton can be given as

3 $$G\left( n \right) = \frac{\beta \Delta P\left( n \right) + \Delta Z\left( n \right) + \alpha Z\left( n \right)}{{\beta P\left( n \right)}}$$

where *n* is the time step number, $\Delta P\left( n \right) = P\left( {n + 1} \right) - P\left( n \right)$ and $\Delta Z\left( n \right) = Z\left( {n + 1} \right) - Z\left( n \right)$; *P*(*n*) and *Z*(*n*) are the phytoplankton and zooplankton abundances, respectively.

It can be seen from (3) that the growth function *G*(*n*) depends not only on *P*(*n*), but also on *P*(*n* + 1). This is due to the fact that *G*(*n*) determines the change in phytoplankton biomass from the time step *n* to the time step *n* + 1. Since phytoplankton consumption by zooplankton can cause changes in zooplankton biomass, *G*(*n*) depends on $\Delta Z\left( n \right).$ In addition, in accordance with (3), when calculating *G*(*n*), we take into account the part of the phytoplankton biomass that was consumed by zooplankton using the term *αZ*(*n*) (Eq. 3).

**Figure 3:**
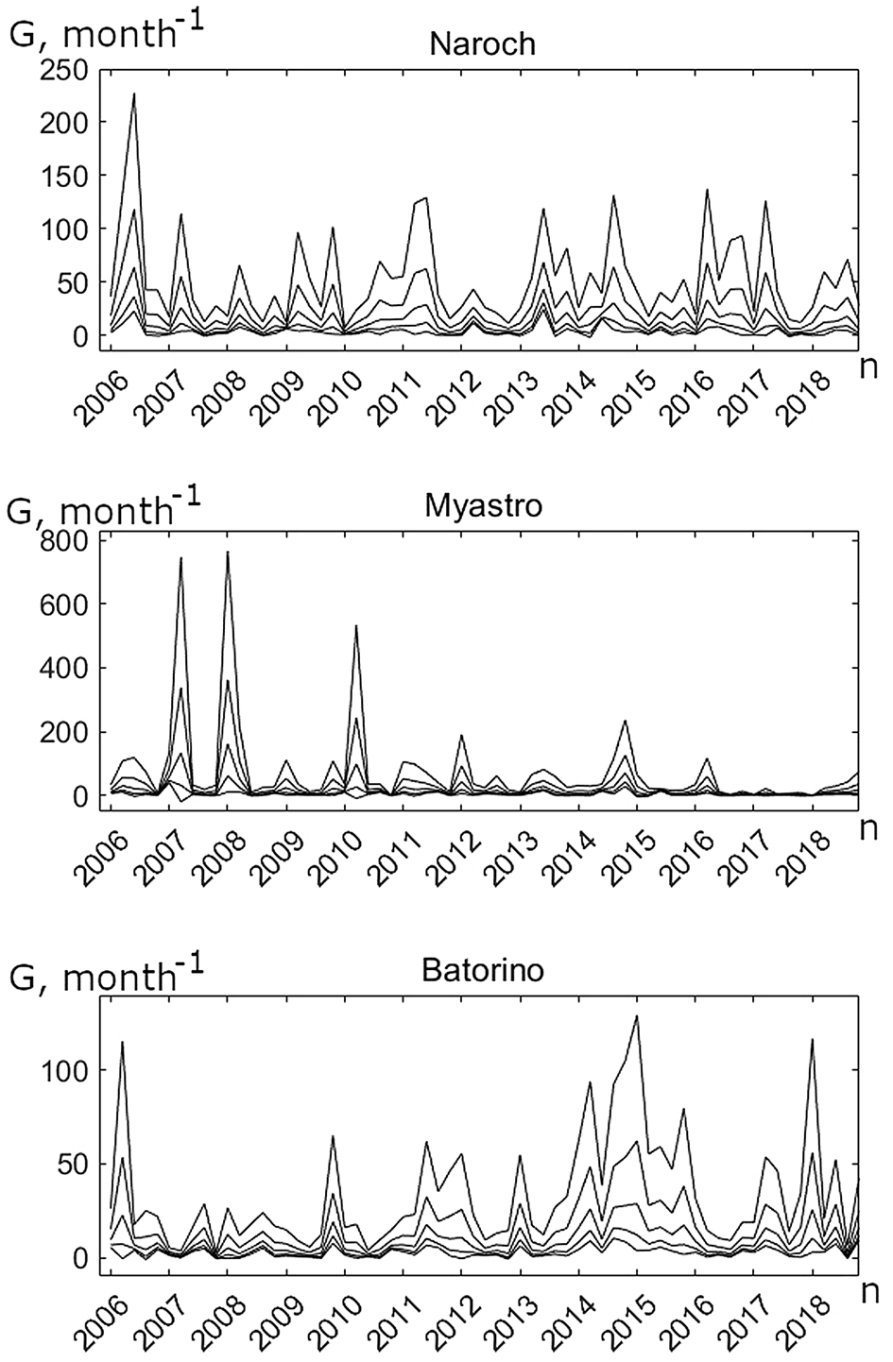
Fluctuations in the phytoplankton growth rate, *G*(*n*), for the Naroch Lakes (time step is 1 month); *α*(month^−1^) = 0.5, 1, 2, 4, 8 (for graphs from bottom to top); *β* = 0.1. The years during which monitoring was carried out are marked under the abscissa axes. During the calculation of the phytoplankton growth rate* G*(*n*), we excluded the cold period of the year for which data were not available. Thus, the calculations excluded the time step between the end of one season and the beginning of the next.

The *G*(*n*) oscillations given by Eq. (3) are shown in Fig. 3. As evident from (3), the growth rate *G*(*n*) depends on two parameters, *α* and *β*. According to Lindeman’s 10% law^25^, the numerical value of *β* should not differ much from 0.1. The numerical values of *α* presented in this paper are within the limits that correspond to the results of monitoring of the Naroch Lakes^26^; *α* ϵ [0, 20] (Fig. 6).

When looking at Fig. 3, the irregular nature of the *G*(*n*) oscillations immediately catches the eye. This irregularity is in good agreement with the chaotic nature of oscillations in the abundance of phytoplankton in the Naroch Lakes^27^. From Fig. 3 it is also seen that an increase in the numerical value of the parameter *α* is accompanied by an increase in the amplitude of the oscillations of the growth function *G*(*n*). In addition, as can be seen from Fig. 3, even with relatively small numerical values of the parameter *α* in the vast majority of cases, i.e., for most values of *n*, *G*(*n*) > 0. This property of the *G*(*n*) function reflects the fact that the oscillatory nature of phytoplankton dynamics persists, despite the occasional drop in the growth rate of phytoplankton to negative values.

**Figure 4:**
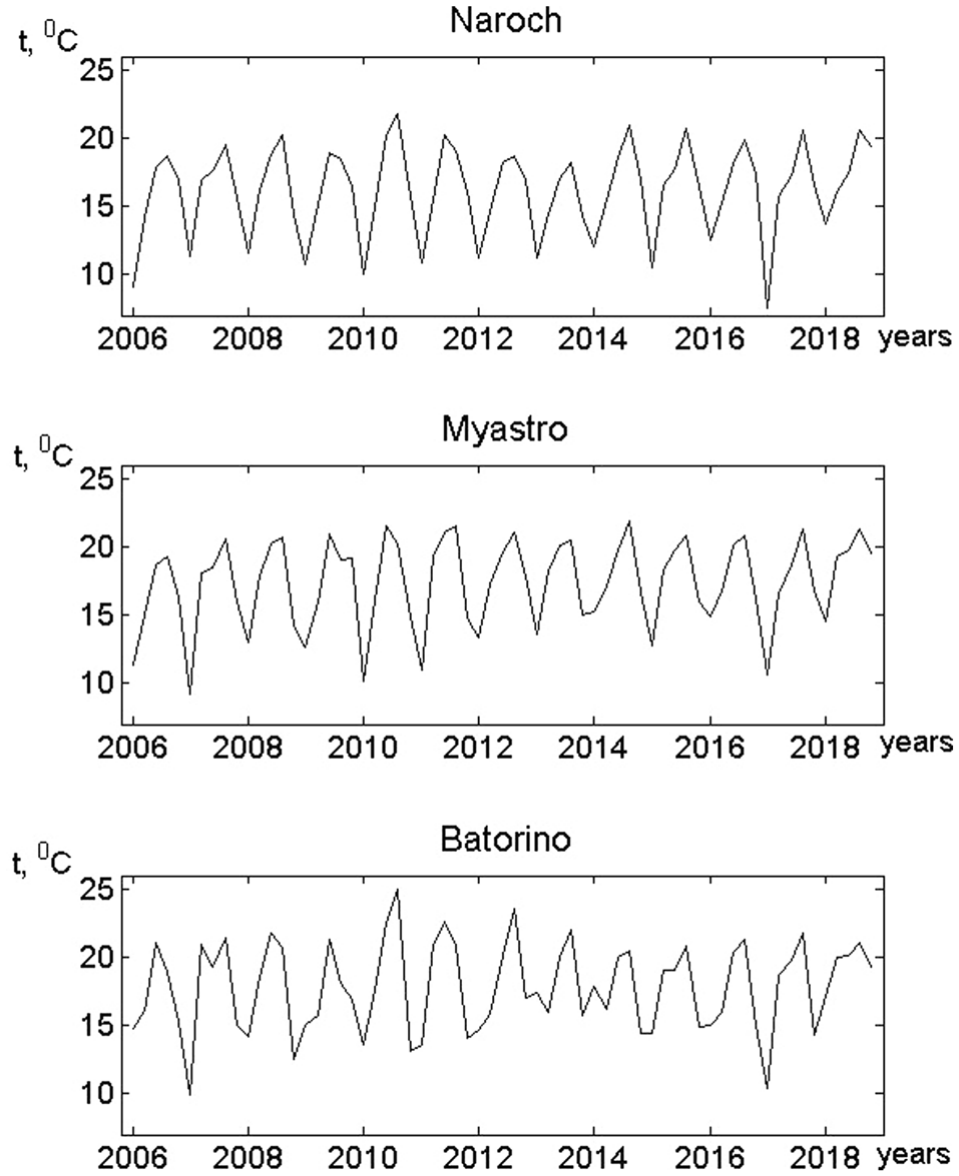
Variations in water temperature (t, °C) obtained during monitoring of the Naroch Lakes (the time step is 1 month). The water temperature measurement were carried out simultaneously with the plankton sampling (see Supporting Materials).

Phytoplankton oscillations (Fig. 1) occur under changing temperature conditions. Figure 4 shows temperature variations in the Naroch Lakes. One can see the almost periodic character of these oscillations, which is the result of seasonality characteristic of the phytoplankton habitats.

**Figure 5:**
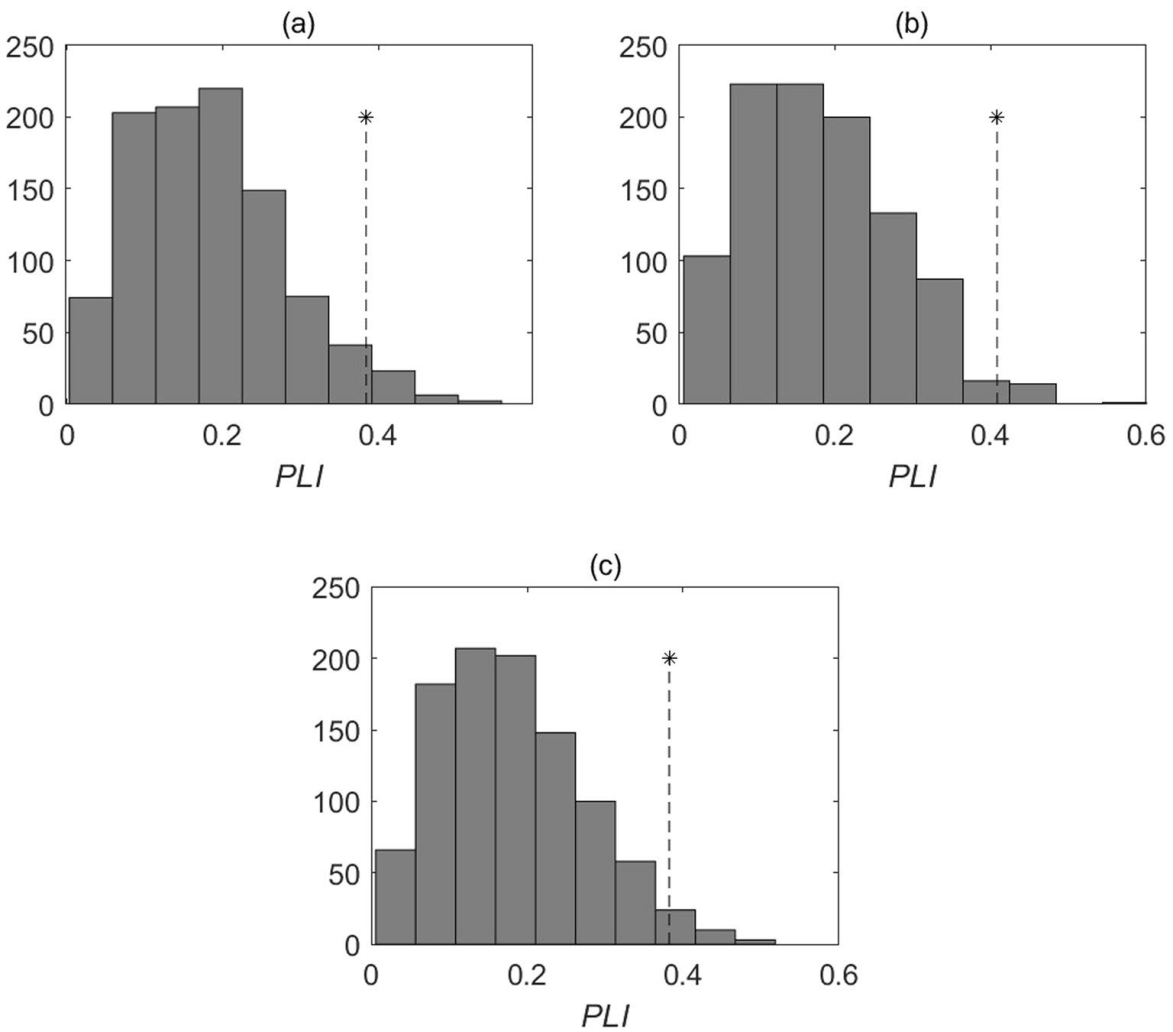
Examples of phase synchronization of phytoplankton growth rate fluctuations and temperature variations in the Naroch Lakes. Values of *PLI* (shown by *) and the distributions of *PLI* values for the surrogate data resulting from 1000 random shuffles of the initial *G*(*n*) (Fig. 3) and temperature (Fig. 4) time series for each of the Naroch Lakes: (**a**) Lake Naroch (*PLI* = 0.38; *α* = 1; *β* = 0.1; the significance value *p* = 96.6%); (**b**) Lake Myastro (*PLI* = 0.41; *α* = 8; *β* = 0.1; the significance value *p* = 98.4%); (**c**) Lake Batorino (*PLI* = 0.38; *α* = 3; *β* = 0.1; the significance value *p* = 97.2%).

In order to numerically characterize the degree of connection between fluctuations in the growth rate of phytoplankton and temperature variations, we use the phase-locking index, *PLI*^28^ (also see "Methods"). Statistically significant values of *PLI* allow us to estimate the degree of phase synchronization between the studied oscillatory processes, for example, between the *G*(*n*) time series (Fig. 3) and the time series representing temperature fluctuations (Fig. 4). The minimum value of *PLI* equal to zero corresponds to the absence of synchronization, and the maximum value of *PLI* equal to 1 means complete phase synchronization of the processes under study. If the value of *PLI* is less than one (but not equal to zero), this indicates incomplete, i.e., slightly out of phase, synchronization of oscillatory processes. The examples of the results of the analysis of phase relations between the *G*(*n*) time series at fixed numerical values of the parameters *α* and *β* and the temperature oscillations are shown in Fig. 5, where the *PLI* values characterizing the phase synchronization of the *G*(*n*) time series and the temperature time series are compared with the distributions of *PLI* values for surrogate data. Surrogate data are used to assess the statistical significance of the value of the phase locking index (see "Methods"). The *PLI* value distributions for the surrogate data shown in Fig. 5 are obtained by multiple random shuffling of the initial time series. Such shuffling does not exclude some random phase synchronization. As a result, the *PLI* values corresponding to the surrogate data can be different from zero. However, the statistically significant value of *PLI*, which characterizes, albeit incomplete, but real, and not random, phase synchronization, differs considerably significantly from those *PLI* values that are characteristic of surrogate data. It is usually assumed that significance value of p is no less than 95% for phase-synchronized processes. Note that although the values of *PLI* in Fig. 5 lie within the surrogate data distributions, nevertheless, the values of the corresponding significance values (*p* ≥ 95%) indicate phase synchronization of the *G*(*n*) and temperature time series. This means that the *G*(*n*) oscillations are phase locked by the water temperature variations.

We also calculated non-parametric correlation coefficients between fluctuations in the growth rate of phytoplankton and temperature variations in all the three reservoirs of the ecosystem of the Naroch Lakes. No statistically significant correlations were found between these processes.

**Figure 6:**
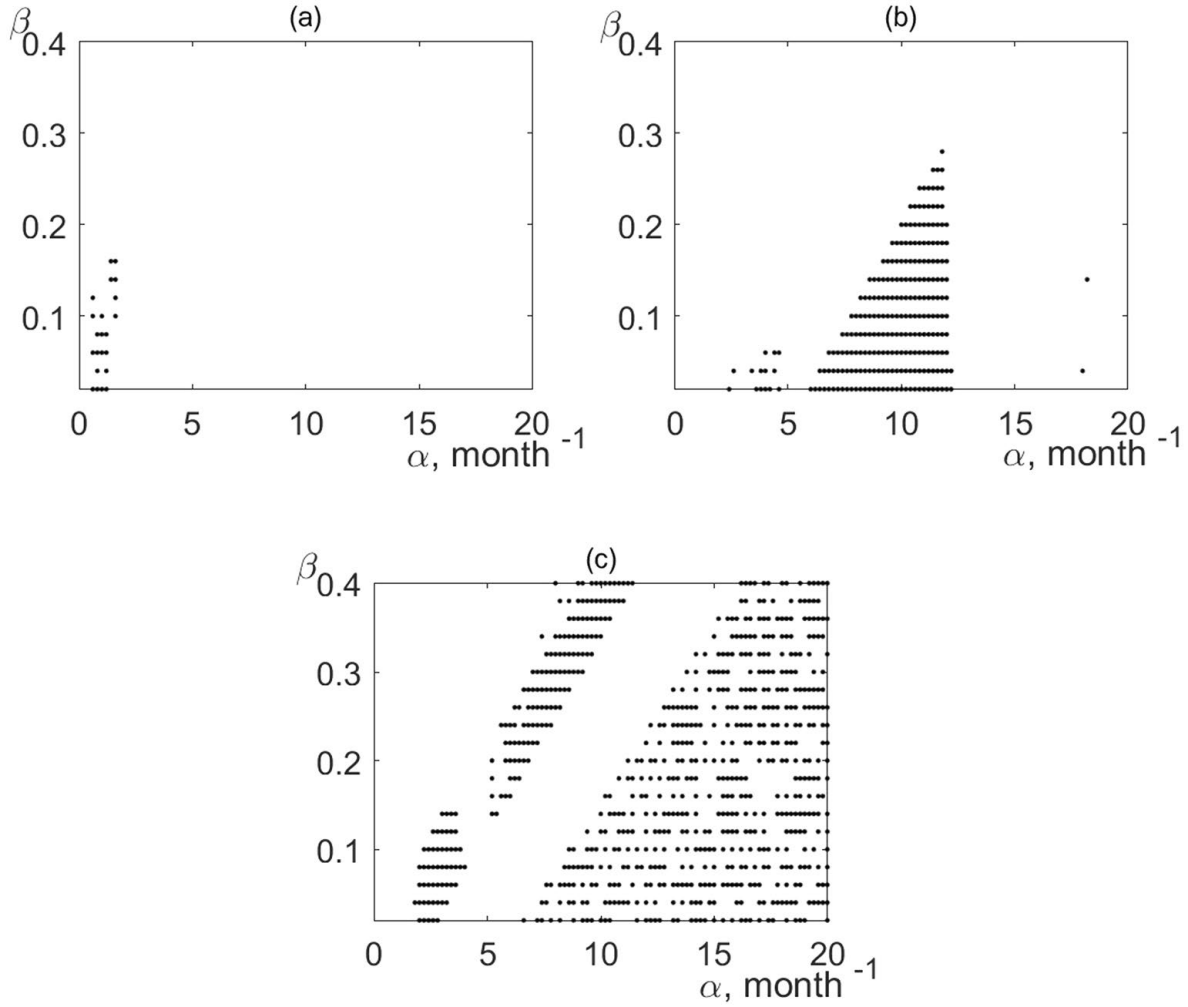
Values of the parameters *α* and* β* (♦), in which the phase synchronization of temperature variations and fluctuations in the growth rate of phytoplankton *G*(*n*) occurs, for: (**a**) Lake Naroch; (**b**) Lake Myastro; (**c**) Lake Batorino.

We performed a sensitivity analysis to study the influence of specific values of the parameters *α* and* β* on the phase synchronization of the *G*(*n*) oscillations and temperature variations (Fig. 6). Lake Batorino, the smallest of the lakes of the Naroch system, is characterized by the largest region in the parameter space (*α*,* β*), in which there is phase synchronization of temperature variations and fluctuations in the growth rate of phytoplankton *G*(*n*). Note that the time-averaged phytoplankton biomass in Lake Batorino is 9.51 mg/l, and this is the largest phytoplankton biomass among the water bodies of the ecosystem of the Naroch Lakes ^29^.

As can be seen from Fig. 6, Lake Naroch, the largest of the reservoirs of the system of the Naroch Lakes, is characterized by the smallest region in the parameter space (α, β), where phase synchronization of temperature fluctuations and fluctuations in the growth rate of phytoplankton *G*(*n*) takes place. By comparison, the time-averaged phytoplankton biomass in Lake Naroch is only 1.09 mg/l^29^. Lake Myastro is characterized by intermediate values of both the phytoplankton biomass (2.72 mg/l^29^) and the size of the region in the parameter space (α, β), where phase synchronization of temperature fluctuations and fluctuations in the growth rate of phytoplankton *G*(*n*) occurs (Fig. 6). We assume that small values of phytoplankton biomass can significantly hinder the synchronization of *G*(*n*) and temperature fluctuations. Note that the increase in the parameter α, which causes an increase in the amplitude of the oscillations of the function *G* (as in Fig. 3) and also may lead to a decrease in the abundance of zooplankton (Eq. 2), can cause a violation of phase synchronization (Fig. 6). The reason for the decrease in the abundance of zooplankton can be both natural mortality and death as a result of trophic interactions. In addition, an increase in the numerical value of the parameter α, which can disrupt the phase synchronization of phytoplankton dynamics and temperature fluctuations (Fig. 6), may also be due to the influence of abiotic factors.

## Discussion

We introduce a new methodology, knowledge-and-data-driven (KDD) modeling. In the context of this methodology, the time series obtained during experiments or observations is directly introduced into mechanistic ecological models. As a result, it becomes possible to identify the functional dependencies that determine the character of the observed fluctuations in population abundance. This is the difference between KDD modeling and the KD models, where these functional dependencies were assumed (often without sufficient grounds) to be known a priori. KDD modeling as a methodology also differs significantly from DD modeling, the results of which are often difficult to interpret.

We used the time series (Figs. 1, 2) from long-term monitoring of the Naroch Lakes^29^ and identified the response of phytoplankton growth rate *G*(*n*) (Fig. 3) to temperature variations (Fig. 4). Namely, we established that fluctuations in the growth rate of phytoplankton can synchronize in phase with temperature variations. It should be noted that phase synchronization of oscillatory processes is a nonlinear phenomenon that is widespread at various levels of biological organization, ranging from cells to populations and communities^30,31^. In this regard, it may be of interest that synchronized cell cycles of unicellular phytoplankton can cause oscillations at the population level^32^. Many studies have been devoted to phase synchronization in ecological systems^33,34^. Although synchronized oscillatory processes are widely represented in ecological systems^35^, their causes and their biological significance in many cases still remain an enigma. Nevertheless, there are examples where the functional role of phase synchronization has been convincingly demonstrated. In particular, it has been shown that phase synchronization can increase the probability of reproductive success, as well as contribute to protection from predation^34,36,37^. Biological and ecological oscillatory processes can be controlled by both endogenous and exogenous factors. For example, exogenous environmental factors such as exposure to light and temperature can significantly affect circadian rhythms^35,38^. The growth rate of phytoplankton can also significantly depend on temperature. In particular, it has been shown that the phytoplankton growth rate monotonically increases with an increase of temperature from 6 °C to 33 °C^39^. However, the relationship between temperature variations and fluctuations in the growth rate of phytoplankton still remains poorly understood due to the fact that temperature variations affect a whole range of physiological processes that underlie the growth of phytoplankton^40^. In the context of our study, we assume that the phase locking of fluctuations in the growth rate of phytoplankton to temperature changes may allow phytoplankters to adjust their physiological status in response to such changes. Phase synchronization of fluctuations in the growth rate of phytoplankton and temperature variations depends on the parameters* α* and* β*. It is worth noting that the lack of the phase synchronization of temperature variations and fluctuations in the growth rate of phytoplankton (white regions in Fig. 6) does not necessarily imply that there is no impact of temperature on phytoplankton growth. In such cases the impact of other factors, such as nutrient fluctuations and/or variations of light level, may have a stronger effect on the phytoplankton dynamics than the temperature variations.

**Figure 7:**
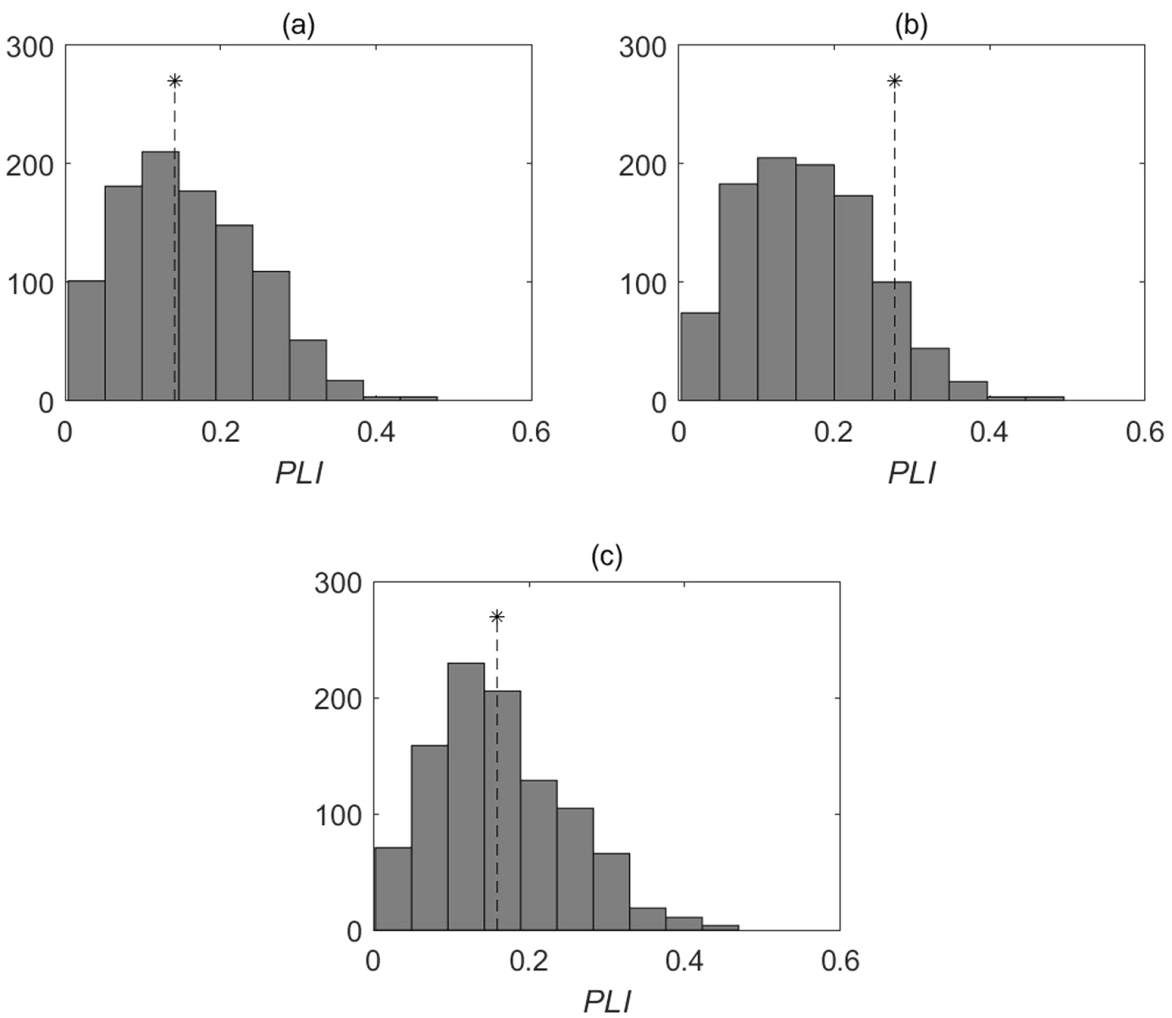
Values of *PLI* (shown by *) and the distributions of *PLI* values for the surrogate data resulting from 1000 random shuffles of the original phytoplankton (Fig. 1) and temperature (Fig. 4) time series for each of the Naroch Lakes: (**a**) Lake Naroch (*PLI* = 0.14; the significance value *p* = 42.8%); (**b**) Lake Myastro (*PLI* = 0.28; the significance value *p* = 89.7%); (**c**) Lake Batorino (*PLI* = 0.16; the significance value *p* = 49.4%).

It follows from Eq. (3) that the fluctuations of phytoplankton *P*(*n*) and zooplankton *Z*(*n*) directly affect the dynamics of the growth rate of phytoplankton. In this regard, the question arises whether temperature variations affect both the fluctuations of phytoplankton and the growth rate of phytoplankton. In other words, does the phase synchronization of *G*(*n*) and temperature fluctuations lead to the occurrence of phase synchronization of temperature and* P*(*n*) fluctuations? It turns out that, unlike the dynamics of the phytoplankton growth rate, fluctuations in the phytoplankton abundance in the Naroch Lakes are not phase-synchronized with temperature fluctuations (cf. Figures 5 and 7). The lack of synchronization between phytoplankton fluctuations and temperature fluctuations does not necessarily mean that there is no influence of temperature on the dynamics of phytoplankton. This may simply mean that phytoplankton dynamics are influenced by a whole number of environmental factors, including, in addition to temperature, nutrient requirements, as well as trophic interactions^41–43^.

Note that the effects of environmental factors and a variety of trophic interactions, with the exception of interactions between phytoplankton and zooplankton, are not directly included in the model (1)–(2). Such effects are taken into account by the model (1)–(2) indirectly, since all trophic interactions in the lake ecosystem, as well as the influence of environmental factors, are reflected in the characteristics of the time series *P*(*n*) and *Z*(*n*). As a result, the dynamics of the growth rate of phytoplankton *G*(*n*), given by Eq. (3), also reflects the influence of all the above factors. Taking into account this circumstance, the degree of phase synchronization between temperature variations and the time series *G*(*n*) and *P*(*n*) (or the absence of such synchronization) given by the numerical value of *PLI*, can be considered as a holistic characteristic of the ecological processes under study. It is worth noting that the numerical evaluation of *PLI* as a holistic parameter, which gives an idea of synchronization of chlorophyll and total phosphorus oscillations, has recently allowed us to characterize the transformation of the ecosystem of the Naroch Lakes as a whole, which occurred in the late 1980s, without resorting to the study of complex interactions of various factors involved in this transformation^24^.

The knowledge-and-data-driven, KDD, modeling that involves direct input of monitoring data into a mechanistic ecological model offers a way to identify some characteristic features of the dynamics of natural, rather than model ecological processes, including those processes (for example, fluctuations in the growth rate of phytoplankton) that were not observed during monitoring of the ecosystem under study. The KDD approach can be a useful addition to those methods of mathematical modeling that have been so far used^9,10^ in studying the mechanisms underlying the functioning of natural ecosystems.

The results presented here, which relate to fluctuations in the phytoplankton growth rate *G*(*n*), give an idea of the dynamics of phytoplankton as a whole, without taking into account the species composition of phytoplankton. A possible future development regarding the study of the contribution of the dynamics of individual phytoplankton species to *G*(*n*) fluctuations may be of significant interest.

It should be noted that the model (1)–(2) does not contain spatial variables. When evaluating *PLI*, we also do not take into account spatial effects. This means that we consider the water bodies of the ecosystem of the Naroch Lakes as polymictic and homothermic reservoirs. They really are, being highly susceptible to wind mixing^8,29^. However, in other reservoirs, consideration of spatial effects in the framework of the KDD modeling may be necessary.

When estimating the numerical values of *PLI*, we average the impact of the phase difference between the time series over time and thus consider the time series as integral objects, actually excluding time (the number of time steps) from consideration. However, taking into account the incompleteness of our knowledge about the behavior of complexly organized aquatic ecosystems, we can expect that the subsequent accumulation of data and, consequently, the lengthening of time series (an increase in *n*) may lead to clarification or even a change in our ideas about the functioning of aquatic communities.

## Methods

### Missing-data imputation

The time series studied contained an insignificant amount of missing data (see Supporting Materials). The missing values were imputed by seasonally splitted missing value imputation (with interpolation) using the impute TS package^44,45^. The imputation algorithm splits the time series into seasons and afterwards performs imputation separately for each of the resulting time series datasets (each containing the data for one specific season). The time series obtained as a result of the imputation are shown in Figs. 1 and 3.

### The analysis of phase relations between time series

For *P*(*n*), *Z*(*n*), *G*(*n*) and temperature oscillations (Figs. 1, 2, 3 and 4) the phase of oscillations can be defined^35^ as the function

$$\varphi \left( t \right) = 2\pi \left( {m + \frac{{t - t_{k} }}{{t_{k + 1} - t_{k} }}} \right), t_{k} \le t \le t_{k + 1} .$$

Here *t*_*k*_ and *t*_*k*+1_ are the points in time at which the oscillations under study reaches their maximums.

To assess the degree of synchronization of two oscillatory processes, a measure of synchronization, the phase-locking index (*PLI*) was suggested^28,46^. It is defined as

$$PLI = \left| {\frac{1}{N}\mathop \sum \limits_{j = 0}^{N - 1} e^{i\Delta \varphi \left( j \right)} } \right|,$$

where *N* is the number of measurements, and Δ*φ* is the phase difference between oscillatory processes. *PLI* is restricted to the interval [0, 1] and reaches 1 if and only if the time series are strictly synchronized, whereas for unsynchronized time series (i.e., for a uniform distribution of Δ*φ*) *PLI* = 0. In real data, neither of these extreme values can be observed, but values between 0 and 1 are typical. Statistical significance testing must be done to establish whether a *PLI* value resulting from the analysis of phase relation between time series indicates a real dynamical coupling between the processes under study. Testing with surrogate data^47^ allows estimating how much synchronized the processes are.

## Supplementary Information

Supplementary Information.

## Data Availability

All data generated or analyzed during this study are included in this published article (and in the supplementary information file). No experiments on plants or animals were carried out.

## References

[1] Solé RV, Bascompte J (2006). Self-Organization in Complex Ecosystems.

[2] Scheffer M (2009). Critical Transitions in Nature and Society.

[3] Lomas MW, Trice TM, Glibert PM, Bronk DA, Mc Carthy JJ (2002). Temporal and spatial dynamics of urea uptake and regeneration rates and concentrations in Chesapeake Bay. Estuaries.

[4] Brown JH, Gillooly JF, Allen AP, Savage VM, West GB (2004). Towards a metabolic theory of ecology. Ecology.

[5] Cross WF, Hood JM, Benstead JP, Huryn AD, Nelson D (2015). Interactions between temperature and nutrients across levels of ecological organization. Glob. Change Biol..

[6] Heinle DR (1969). Temperature and zooplankton. Chesap. Sci..

[7] Moore MV, Folt CL, Stemberger RS (1996). Consequences of elevated temperatures for zooplankton assemblages in temperate lakes. Arch. Hydrobiol..

[8] Medvinsky AB (2017). Temperature as a factor affecting fluctuations and predictability of the abundance of bacterioplankton. Ecol. Complex..

[9] Kot M (2001). Elements of Mathematical Ecology.

[10] Otto SP, Day T (2007). Biological Guide to Mathematical Modeling in Ecology and Evolution.

[11] Smith WO, Ainley DG, Cattaneo-Vietty R (2007). Trophic interactions within the Ross Sea continental shelf ecosystem. Philos. Trans. R. Soc. B Biol. Sci..

[12] Trites AW (2019). Marine mammal trophic levels and trophic interactions. Encicl. Ocean Sci..

[13] Maurer BA (1999). Untangling Ecological Complexity: The Macroscopic Perspective.

[14] Medvinsky AB (2019). Population dynamics: Mathematical modeling and reality. Biophysics.

[15] Viseconti M, Doblaré M, Merodio J (2015). Multiscale modeling of human pathophysiology. Biomathematics.

[16] Sugihara G, May RM (1990). Nonlinear forecasting as a way of distinguishing chaos from measurement error in time series. Nature.

[17] Munch SB, Rogers T, Sugihara G (2022). Recent developments in empirical dynamic modelling. Methods Ecol. Evol..

[18] Jennings S, Kaiser M, Reynolds JD (2001). Marine Fisheries Ecology.

[19] Haykin S (1998). Neural Networks: A Comprehensive Foundation.

[20] Poli R, Langdon WB, McPhee NF (2008). A Field Guide to Genetic Programming.

[21] Solomatine D, See LM, Abrahart RJ, Abrahart RJ, See LM, Solomatine DP (2008). Data-driven modeling: Concepts, approaches and experiences. Practical Hydroinformatics Computational Intelligence and Technological Developments in Water Approaches.

[22] Turchin P (2003). Complex Population Dynamics: A Theoretical/Empirical Synthesis.

[23] Adamson MW, Morozov AY (2013). When can we trust our model predictions? Unearthing structural sensitivity in biological systems. Proc. R. Soc. A..

[24] Rusakov AV (2022). Phase synchronization of chlorophyll and total phosphorus oscillations as an indicator of the transformation of a lake ecosystem. Sci. Rep..

[25] Lindeman RL (1942). The trophic-dynamic aspect of ecology. Ecology.

[26] Winberg GG, Pechen GA, Shushkina EA (1965). Production of plankton crustaceans in three lakes of different types. Zool. Zhurnal.

[27] Medvinsky AB (2015). Chaos far away from the edge of chaos: A recurrence quantification analysis of plankton time series. Ecol. Complex..

[28] Kuramoto Y (1984). Chemical Oscillations, Waves, and Turbulence.

[29] Adamovich BV (2017). Eutrophication, oligotrophication, and benthification in Naroch Lakes: 40 years of monitoring. J. Sib. Fed. Univ..

[30] Glass L, Mackey MC (1988). From Clocks to Chaos: The Rhythms of Life.

[31] Glass L (2001). Synchronization and rhythmic processes in physiology. Nature.

[32] Massie TM, Blasius B, Weithoff G, Gaedke U, Fussmann GF (2010). Cycles, phase synchronization, and entrainment in single-species phytoplankton populations. Proc. Natl. Acad. Sci. U. S. A..

[33] Buck J, Buck E (1968). Mechanism of rhythmic synchronous flashing of fireflies. Scince.

[34] Woodland DJ, Cabanban AS, Taylor VM, Taylor RJ (2002). A synchronized rhythmic flashing light display by schooling Leiognathus splendens (Leiognathidae: Perciformes). Mar. Freshw. Res..

[35] Pikovsky A, Rosenblum M, Kurths J (2001). Synchronization A Universal Concept in Nonlinear Sciences.

[36] Greenfield MD (1994). Synchronous and alternating choruses in insects and anurans: Common mechanisms and diverse functions. Am. Zool..

[37] Legett HD, Page RA, Bernal XE (2019). Synchronized mating signals in a communication network: The challenge of avoiding predators while attracting mates. Proc. R. Soc. B Biol. Sci..

[38] Winfree AT (1980). The Geometry of Biological Time.

[39] Pulsifer J, Laws E (2021). Temperature dependence of freshwater phytoplankton growth rates and zooplankton grazing rates. Water.

[40] Edwards KF, Thomas MK, Klausmeier ChA, Litchman E (2016). Plankton growth and the interaction of light and temperature: A synthesis at the species and community level. Limnol. Oceanogr..

[41] López-Flores R, Boix D, Badosa A, Brucet S, Quintana XD (2009). Environmental factors affecting bacterioplankton and phytoplankton dynamics in confined Mediterranean salt marshes (NE Spain). J. Exp. Mar. Biol. Ecol..

[42] Nowrouzi S, Valavi H (2011). Effects of environmental factors on phytoplankton abundance and diversity in Kaftar Lake. J. Fish. Aquat. Sci..

[43] Cao J, Chu Z, Du Y, Hou Z, Wang S (2016). Phytoplankton dynamics and their relationship with environmental variables of Lake Poyang. Hydrol. Res..

[44] Steffen M, Bartz-Beielstein T (2017). impute TS: Time series missing value imputation in R. R J..

[45] R Core Team (2020). R: A Language and Environment for Statistical Computing.

[46] Mormann F, Lehnertz K, David P, Elger CE (2000). Mean phase coherence as a measure for phase synchronization and its application to the EEG of epilepsy patients. Phys. D..

[47] Kantz H, Schreiber T (1997). Nonlinear Time Series Analysis.

