# Supplementary material for: Direct input of monitoring data into a mechanistic ecological model as a way to identify the phytoplankton growth-rate response to temperature variations
Source: Sci Rep. 2023 Jun 22;13:10124. doi: 10.1038/s41598-023-36950-3 (PMC10287759; doi:10.1038/s41598-023-36950-3)
Supplement: Supplementary file 1 — Supplementary Information. [file 41598_2023_36950_MOESM1_ESM.pdf]

Supporting Materials

1. Study site

The system of the Naroch Lakes is located in the northwestern Belarus in the Neman River basin and includes three water bodies: Lake Batorino, Lake Myastro, and Lake Naroch. These water bodies are interconnected by channels. The main characteristics of the Naroch Lakes are given in Table 1. The Naroch Lakes are completely frozen for much of the period from the late November to April.

Table 1. The main characteristics of the lakes

Characteristics	Lake Naroch	Lake Myastro	Lake Batorino
Surface area, km^2	79,6	13,1	6,3
Water volume, millions m^3	710,0	70,1	18,7
Depth (mean/maximum), m	8,9/24,8	5,4/11,3	2,4/5,5
Total water exchange (years)	10–11	2.5	1.0
Trophic state	Oligotrophic-mesotrophic	mesotrophic	eutrophic

2. Field sampling

Water samples were collected monthly at specific monitoring points at the pelagic zones of the lakes during the vegetative season (from May to October) with the use of the two-liter Ruttner sampler. The samples were collected from six different depths (0.5, 3, 6, 8, 12 and 16 m) in Lake Naroch(54°53'10.56"N, 26°43'12.12"E), four depths (0.5, 4, 7 and 9 m) in Lake Myastro (54°52'0.91"N, 26°52'49.86"E), and three depths (0.5, 3 and 5 m) in Lake Batorino (54°50'47.94"N, 26°58'3.36"E). The water samples from all the depths were mixed in such a way that the water volume of each level (depth) in the mixed sample was proportional to the fraction of the level in the total water volume in each of the lakes according to bathymetry. At the same depths, water temperature was measured using a mercury deep-water thermometer with a scale resolution of 0.1°C.

3 Phyto- and zooplankton

Samples of 0.5 liters for phytoplankton and 10 liters for zooplankton measurements were taken out from each of the mixed samples. Phytoplankton samples were fixed with Utermöhl's solution [1]. Then the samples were sedimented in total darkness for not less than one week. These samples were concentrated very accurately up to 100–150 ml by pouring off all excess water with the use of thin silicon or rubber siphon covered with two-layer silk sieve with 77 μm mesh. The resulting concentrate was poured into plankton bottles and settled for no less than 2 – 3 days. Then the extra water was drawn off using a medical syringe with thin vinyl tubule at its end. Finally, the actual sample volume was 15 – 30 ml depending on the sediment thickness. The light Zeiss Axiolab microscope was used to analyze phytoplankton samples. The phytoplankton abundance is expressed in cell number (number of one-celled species, number of cells in filaments and colonies) per liter. A Fuchs-Rosenthal chamber with a volume of 3.2 mm^3 was used to count small phytoplankton individuals. The larger phytoplankton such as *Ceratium, Asterionella, Melosira, Aulacoseira, Tabellaria, Fragilaria, Microcystis, Coelosphaerium,*

Anabaena, and some others were counted with the use of a 1 ml chamber while large colonial organisms (*Gloeotrichia echinulata*, *Volvox*) were counted with the use of a Bogorov chamber

Estimation of phytoplankton biomass was carried out by “the Kiselev’s method of true volumes” [2]. Using this method, cell or organism volumes were calculated for each species by applying a solid geometric shape (ellipsoid, sphere, rod, cone, etc.) most closely matching with the shape of the cells or organisms [3, 4]. The relative density of phytoplankton was set to 1. The total biomass of phytoplankton was calculated by taking the sum of the biomass of each isolated individual.

The zooplankton samples were filtered using Apstein plankton net with 64 μm mesh size. Resulting sediment with a volume of 150 ml was poured into a plastic bottle filled with 4% formalin and it settled for 10 days. Then, the sample volume was reduced to 50 ml by decanting of top layer without stirring-up of sediment. The silicon siphon covered with 55 μm mesh net was used for the decanting. With a pipette dispenser, 2 – 6 ml of the sample was taken off for analysis. Counting and zooplankton species identification were carried out with two repetitions using microscopes Zeiss Axiolab and Zeiss Stemi 2000. The number of zooplankton individuals was counted in 40 mm Petri dish with the net pattern plotted on the bottom. Zooplankter sizes were measured with the help of an ocular micrometer. The body mass of *Cladocera* and *Copepoda* was determined using the equations which that describe exponential dependencies between length and body volume [5]. The body shapes of *Rotifera* were compared with the defined geometric figures. The body volume was determined according to geometrical equations [5]. The relative density of zooplankton was set to 1. The total biomass of zooplankton was calculated by taking the sum of the biomass of each isolated individuals. Raw data of phyto- and zooplankton were descripted for 2006-2013 in [6] and for 2014-2018 in [7-11].

Additional references

1. Mikheyeva, T.M. Methods of quantitative accounting of nanophytoplankton (review). Hydrobiological Journal 25, 3-21 (1989) (in Russian).
2. Kiselev, I.A. Plankton of seas and continental reservoirs (Nauka, Leningrad) (1969) (in Russian).
3. Hillebrand, H., Dürselen, C.-D., Kirschtel, D. Pollingher, U., Zohary, T. Biovolume calculation for pelagic and benthic microalgae. Journal of. Phycology 35, 403–424 (1999)
4. Mikheyeva, T.M. Algal flora of Belarus. Taxonomic catalogue (BSU, Minsk) (1999) (in Russian)
5. Balushkina, E.V., Vinberg, G.G. The relationship between zooplankter weight and body length. In: General foundations for the study of aquatic ecosystems (Nauka, Leningrad) pp. 169-172 (1979) (in Russian)
6. Medvinsky, A.B., Adamovich, B.V., Chakraborty, A., Lukyanova, E.V., Mikheyeva, T.M., Nurieva, N.I., Radchikova, N.P., Rusakov, A.V., Zhukova, T.V. Chaos far away from the edge of chaos: A recurrence quantification analysis of plankton time series. Ecological Complexity 23, 61-67 (2015).
7. Mikheeva, T.M. (Ed.). Bulletin of the Ecological State of Lakes Naroch, Myastro, Batorino (2014). Beloruss. Gos. Univ., Minsk (2015) (in Russian).
8. Mikheeva, T.M. (Ed.). Bulletin of the Ecological State of Lakes Naroch, Myastro, Batorino (2015). Beloruss. Gos. Univ., Minsk (2016) (in Russian).
9. Mikheeva, T.M. (Ed.). Bulletin of the Ecological State of Lakes Naroch, Myastro, Batorino (2016). Beloruss. Gos. Univ., Minsk (2017) (in Russian).
10. Mikheeva, T.M. (Ed.). Bulletin of the Ecological State of Lakes Naroch, Myastro, Batorino (2017). Beloruss. Gos. Univ., Minsk (2018) (in Russian).

11. Mikheeva, T.M. (Ed.). Bulletin of the Ecological State of Lakes Naroch, Myastro, Batorino (2018). Beloruss. Gos. Univ., Minsk (2019) (in Russian).

Below we present the results of field measurements, which were carried out in 2006 – 2018 in the Naroch Lakes.

PhytoN – the phytoplankton biomass (mg/l) in Lake Naroch..

PhytoM - the phytoplankton biomass (mg/l) in Lake Myastro.

PhytoB – the phytoplankton biomass (mg/l) in Lake Batorino.

ZooN – the zooplankton biomass (mg/l) in Lake Naroch.

ZooM – the zooplankton biomass (mg/l) in Lake Myastro.

ZooB – the zooplankton biomass (mg/l) in Lake Batorino.

TempN – temperature in Lake Naroch (^{0}C).

TempM – temperature in Lake Myastro (^{0}C).

TempB – temperatute in Lake Batorino (^{0}C).

Year	Month	PhytoN	PhytoM	PhytoB	ZooN	ZooM	ZooB	TempN	TempM	TempB
2006	5	1,36	3,36	4,66	0,62		1,29	9,10	11,35	14,80
2006	6	0,46	2,30	2,20	0,75		3,41	14,22	15,08	16,07
2006	7	0,33	2,47	6,30	0,90	4,01	1,15	17,84	18,70	21,10
2006	8	1,88	1,56	8,07	1,05	1,45	2,81	18,67	19,33	18,97
2006	9	1,24	8,12	3,97	0,71	0,73	0,93	16,93	16,33	15,23
2006	10	0,63	3,64	1,54				14,49	11,50	9,10
2007	5	2,36	0,76	5,41	0,51	0,93	0,29	11,31	9,15	9,87
2007	6	0,48	0,35	8,59	0,70	3,60	0,42	16,87	18,08	20,97
2007	7	1,21	2,37	3,64	0,47	0,92	0,62	17,60	18,50	19,27
2007	8	3,21	4,76	4,42	0,57	1,17	1,42	19,53	20,63	21,43
2007	9	1,00	2,26	22,40	0,35	0,99	1,00	15,56	15,95	15,00
2007	10	1,17	1,41	10,14	0,31	0,51	0,38	11,42	12,13	10,77
2008	5	0,67	0,33	8,40	0,14	3,33	3,00	11,51	13,05	14,13

2008	6	0,34	0,73	8,14	0,26	2,00	1,31	16,17	17,90	18,63
2008	7	1,02	7,98	3,66	0,33	1,12	0,78	18,87	20,28	21,77
2008	8	2,52	1,86	4,17	0,43	0,61	1,05	20,22	20,73	20,70
2008	9	0,76	1,56	9,55	0,36	0,42	2,09	14,37	14,28	12,57
2008	10	0,43	1,19	4,53	0,35	1,37	1,95	11,10	10,50	10,00
2009	5	0,97	1,72	12,57	0,08	2,50	2,32	10,73	12,60	15,00
2009	6	0,56	3,34	16,71	0,69	1,49	1,90	15,11	15,95	15,70
2009	7	0,84	7,63	20,66	0,55	1,19	1,48	18,90	20,48	21,30
2009	8	1,80	5,84	11,95	0,57	1,35	2,03	18,52	19,00	18,10
2009	9	0,69	1,27	2,09	0,93	1,84	1,60	16,48	19,13	16,93
2009	10		1,66	3,97		0,90	2,19	10,59	6,63	4,60
2010	5	2,94	2,38	9,85	0,24	0,79	1,92	10,07	10,10	13,53
2010	6	1,79	0,43	9,54	0,53	3,08	2,30	14,87	16,08	17,63
2010	7	1,44	2,26	12,77	0,58	0,97	0,58	20,28	21,52	22,47
2010	8	1,02	3,39	9,17	0,93	1,13	1,19	21,83	22,18	25,02
2010	9	0,90	37,89	5,19	0,58	0,70	0,71	15,72	14,90	13,10
2010	10	1,34	4,36	16,21	0,72	1,39	1,57	10,05	10,90	9,40
2011	5	1,92	1,55		1,27	2,05	1,44	10,81	10,98	13,60
2011	6	1,10	1,51		1,80	2,00	2,30	15,45	19,35	21,00
2011	7	0,64	0,96		1,07	0,81	2,72	20,29	21,03	22,67
2011	8	1,40	2,67		0,70	1,04	3,28	19,07	21,56	20,83
2011	9	1,60	14,91		0,33	2,29	5,93	16,03	14,78	14,12
2011	10	1,18	2,75		0,21	2,05	3,41	11,75	8,50	6,20
2012	5	0,84	0,88	5,25	0,30	2,16	3,92	11,22	13,35	14,63
2012	6	0,47	3,07	5,72	0,20	1,43	1,63	14,65	17,25	15,87
2012	7	1,66	3,61	10,03	0,53	0,90	1,11	18,16	19,65	20,10
2012	8	2,19	3,24	10,34	0,60	2,50	1,60	18,66	21,08	23,57
2012	9	2,87	9,16	10,77	0,36	2,01	2,20	16,97	17,88	17,07

2012	10	1,11	10,38	6,39	0,98	1,98	0,55	13,35	12,50	13,30
2013	5	2,12	5,41	3,36	0,73	0,69	2,18	11,25	13,60	17,36
2013	6	0,63	1,59	10,91	0,41	1,23	2,43	14,45	18,18	
2013	7	0,41	2,04	11,83	0,52	1,80	1,76	17,03	20,08	
2013	8	1,52	4,84	7,44	1,15	3,81	2,49	18,20	20,45	
2013	9	0,56	6,23	6,42	0,56	2,11	2,70	14,30	15,00	
2013	10	0,85	3,95	2,81	0,60	1,18	2,28	10,00	8,13	
2014	5	1,79	3,38	3,98	0,55	1,38	3,05	12,08	15,28	17,90
2014	6	1,06	2,44	2,94	0,86	0,97	3,34	15,48	17,10	16,17
2014	7	0,84	1,60	7,92	0,26	0,45	3,79	18,62	19,78	19,97
2014	8	0,87	1,70	3,87	1,47	2,48	4,24	20,93	21,90	20,53
2014	9	1,40	0,78	4,68	1,15	2,17	6,03	16,87	16,70	14,47
2014	10	1,02	1,22	2,10	1,07	3,30	7,22	12,02	9,78	8,70
2015	5	1,25	6,31	3,56	0,61	5,75	5,97	10,55	12,75	14,40
2015	6	2,94	2,93	5,90	0,67	0,95	4,04	16,60	18,30	19,03
2015	7	1,58	1,05	5,99	0,73	0,10	4,28	17,80	19,80	19,03
2015	8	2,88	5,68	8,89	1,18	1,09	5,16	20,75	20,80	20,87
2015	9	1,31	5,26	5,76	0,87	1,16	5,99	16,43	16,00	14,93
2015	10	0,64	7,51	5,94	0,91	0,80	3,94	11,22	8,73	7,43
2016	5	1,78	3,07	4,09	0,45	1,36	1,56	12,52	14,85	15,00
2016	6	0,31	1,03	8,17	0,54	1,49	1,55	15,30	16,83	16,00
2016	7	0,78	6,61	8,39	0,45	1,10	0,99	18,05	20,10	20,37
2016	8	0,78	7,28	11,56	0,89	0,35	1,37	19,93	20,83	21,30
2016	9	0,59	1,98	6,88	0,73	0,34	1,39	17,45	16,20	15,07
2016	10	0,70	1,07	6,45	0,37	0,46	3,24	8,80	9,85	9,03
2017	5	2,44	7,25	6,90		0,20	1,47	7,50	10,64	10,43
2017	6	0,45	4,42	4,52	0,76	1,32	2,86	15,75	16,60	18,67
2017	7	0,77	5,84	7,07	0,35	0,28	4,00	17,32	18,75	19,80

2017	8	2,55	7,38	20,15	0,54	0,66	3,51	20,63	21,33	21,83
2017	9	1,16	6,72	9,42	0,17	0,91	4,29	16,58	16,85	14,27
2017	10	0,85	5,49	6,48	0,28	0,13	2,62	11,60	10,35	9,43
2018	5	2,64	10,25	2,26	0,93	0,09	3,45	13,72	14,60	17,10
2018	6	0,79	2,34	7,65	0,62	0,59	1,81	16,05	19,35	19,97
2018	7	0,71	2,97	5,51	0,37	1,03	3,31	17,45	19,80	20,13
2018	8	0,65	3,18	32,26	0,58	1,63	3,03	20,60	21,30	21,07
2018	9	1,39	2,29	5,51	0,51	2,19	2,44	19,43	19,48	19,33
2018	10	1,21	3,30	3,17	0,33	1,77	6,67	12,13	11,18	10,47
